# Two Cases of Branchial Fistulæ

**Published:** 1893-06

**Authors:** George Parker

**Affiliations:** Assistant-Physician to the Bristol General Hospital


### TWO CASES OF BRANCHIAL FISTULÆ.

BY

George Parker, M.D., M.A. Cantab.,

*Assistant-Physician to the Bristol General Hospital.*

---

J. J., a fairly healthy lad of 19, was attacked by pneumonia during last summer, from which he made a rapid recovery. Whilst attending him I noticed a small opening on the right side of his neck, large enough to admit a fine probe. A little muco-purulent fluid exuded from it, especially if slight pressure was applied on the side nearer the head. The orifice was seen to be the termination of a tube, about the size of a small quill, which could be felt under the skin, running upwards and backwards towards the right corner of the hyoid bone. The opening was near the front edge of the sterno-mastoid, about 2 inches above the clavicle, in what would be the position of the cleft between the thyro-hyoid and sub-hyoid arches. On the left side a slight dimple was noticed on a corresponding spot, and another rather higher up along the line of the sterno-mastoid; but no orifice or tube could be made out. The fistula gives no trouble, and has existed from birth, so far as can be learned. Rather more fluid comes from it when the patient has a cold, but nothing escapes from it when he is drinking. No other members of his family have anything like it, nor have they any cleft palate or similar deformities connected with the closure of the branchial arches. No cartilaginous nodules or remnants of a cervical rib can be detected in the walls of the fistula. The external ears are well formed, and there are no supernumerary auricles. As we often find in these cases, the patient is slightly deaf; and he has suffered from suppurative otitis.

The second instance occurred in a male infant born in October of last year. Immediately after birth I noticed one or two minute warty growths on the left cheek, which withered away, or were rubbed off, after a few days. In front of the left ear is a soft, fleshy, flattened mass, probably a supernumerary auricle, and between it and the commencement of the helix is a small, well-defined pit, sufficient to admit the point of a probe, but shallow. No tube was felt beneath the skin, but the hair, of which the child had a plentiful crop, was absent for a short distance above this ear. In every other respect he was a well-formed boy, weighing 9½ pounds at birth, and he has certainly some power of hearing.

Little or nothing appears to be known of the causes which induce failure of the branchial arches to close perfectly at the beginning of the third month of pregnancy. With the lateral clefts and their non-union are connected fissure of the palate and other deformities. Probably the dermoid cysts of the orbit, cheeks, and neck, as well as branchial and aural fistulæ, have a similar origin. On the other hand, a want of union in the mesial line of the body produces ectopia cordis, or vesicæ, and the rare instances of fistula in the middle line of the trachea. The ordinary branchial fistulæ are always well to one side, and never open into the trachea, as supposed by Dzondi, who first described them in 1829. Many of them terminate in a *cul-de-sac*, but others open into the pharynx. Their most frequent site is immediately above the clavicle in the situation of the fourth cleft, and they are often hereditary. Morbid adhesion of the amnion has been described in some cases. My second case is akin to the rare and incomplete fistulæ situated on the helix itself, described by Paget.[1] In such instances the remains of the cleft are carried upwards by the out-growth of the external ear, the auditory canal being often deficient. In the present case a part of the auricle, with a remnant of the canal, seems to have been pushed forward and detached, while the greater portion has developed in the normal site, so that there is an attempt at duplication of the ear. It is uncertain whether in this child there is defective formation of the internal passages, as usually occurs.

[1] *Medico-Chirurgical Transactions*, vol. lxi. 1878.

